# CRISPR/Cas9-mediated gene knockout in human adipose stem/progenitor cells

**DOI:** 10.1080/21623945.2020.1834230

**Published:** 2020-10-19

**Authors:** Markus Mandl, Heike Ritthammer, Asim Ejaz, Sonja A. Wagner, Florian M. Hatzmann, Saphira Baumgarten, Hans P. Viertler, Marit E. Zwierzina, Monika Mattesich, Valerie Schiller, Tina Rauchenwald, Christian Ploner, Petra Waldegger, Gerhard Pierer, Werner Zwerschke

**Affiliations:** a Division of Cell Metabolism and Differentiation Research, Research Institute for Biomedical Aging Research, University of Innsbruck, Austria; b Center for Molecular Biosciences Innsbruck (CMBI), University of Innsbruck, Austria; c Adipose Stem Cell Center, Department of Plastic Surgery, University of Pittsburgh, PA, USA; d Department of Plastic and Reconstructive Surgery, Innsbruck Medical University, Innsbruck, Austria

**Keywords:** Adipose stem cells, ageing, CRISPR/Cas9, genome editing, loss-of-function, obesity

## Abstract

The CRISPR/Cas9 system is a powerful tool to generate a specific loss-of-function phenotype by gene knockout (KO). However, this approach is challenging in primary human cells. In this technical report, we present a reliable protocol to achieve a functional KO in the genome of human adipose stem/progenitor cells (ASCs). Using Sprouty1 (*SPRY1*) as a model target gene for a CRISPR/Cas9 mediated KO, we particularize the procedure including the selection of the CRISPR/Cas9 target sequences and the employment of appropriate lentiviral vectors to obtain a functional gene KO. The efficiency of CRISPR/Cas9 to mutate the *SPRY1* gene is determined by a PCR-based mutation detection assay and sequence analysis. Effects on mRNA and protein levels are studied by RT-qPCR and Western blotting. In addition, we demonstrate that CRISPR/Cas9 mediated *SPRY1* KO and gene silencing by shRNA are similarly effective to deplete the Sprouty1 protein and to inhibit adipogenic differentiation. In summary, we show a reliable approach to achieve a gene KO in human ASCs, which could also apply to other primary cell types. **Abbreviations:** ASC: Adipogenic Stem/Progenitor Cell; Cas: CRISPR-associated system; CRISPR: Clustered Regularly Interspaced Palindromic Repeat; gDNA: Genomic DNA; GOI: Gene of interest; gRNA: Guide RNA; NHEJ: Non-homologous end joining; Indel: Insertion/Deletion; PAM: Protospacer adjacent motif; sWAT: Subcutaneous white adipose tissue; TIDE: Tracking of indels by decomposition

## Introduction

Obesity is a major public health challenge world-wide [1]. This metabolic disorder is characterized by an excessive increase in white adipose tissue (WAT) mass resulting in metabolic and endocrine dysfunctions [2]. Adipogenic stem/progenitor cells (ASCs) play a pivotal role in adipose tissue homoeostasis and regeneration and are also necessary for the expansion of the adipose tissues in obesity. Increasing studies suggest that the accumulation of senescent ASCs in subcutaneous (s)WAT of obese people leads to exhaustion of the ASC pools already in middle-aged individuals accelerating the establishment of severe obesity-associated comorbidities [3–13]. Our previous studies demonstrated that weight-loss postpones ASC senescence [9–14]. An in-depth analysis of early passages of ASCs isolated from given donors is essential to better understand the effects of obesity and weight-loss on gene expression and function in these cells.

To elucidate the role of a particular gene-of-interest (GOI) in a given cell type the generation of a loss-of-function phenotype is necessary [15]. Disruption or knockout (KO) of a GOI can be achieved by mutating the genomic DNA (gDNA) leading to a frame-shift and the lack of a functional gene product [16,17]. Introducing mutations like insertions or deletions into gDNA is part of modern genome-editing [16]. *Clustered Regularly Interspaced Short Palindromic Repeats* (CRISPR) together with the *CRISPR-associated system* (Cas) 9 enzyme have become a superior technique for determinable gene knockouts in stem cells [16]. Since the seminal publication by *Jinek et al*., 2012 [18], this microbial immune system enabled a novel experimental approach [17]. The Cas 9 enzyme is an RNA-guided endonuclease which is directed to its intended target site in the genomic DNA by Watson-Crick base pairing [16,19]. Thus, by experimentally modifying a synthetic guide RNA (gRNA) which contains a RNA-scaffold for Cas 9-binding and a variable region of approximately 20 base pairs for the formation of the gRNA/DNA heteroduplex, the Cas 9 endonuclease can be directed to a particular genomic loci to cleave the DNA at that site [16,20]. In addition to the interaction between the gRNA sequence and the complementary genomic target DNA, another motif adjacent to the target DNA sequence, referred to as *Protospacer Adjacent Motif* (PAM), is mandatory for the activation of the Cas 9 endonuclease [16,17]. The most widely used CRISPR/Cas9 system is derived from *Streptococcus pyogenes*. This system requires a PAM motif of the nucleotide sequence NGG. The Cas 9 enzyme cleaves the target DNA three base pairs upstream of the NGG motif to generate the DNA double strand break (DSB) [16,17]. DSBs are lethal to the cell and must be repaired. In eukaryotic cells DSBs are repaired by one of two mechanisms, homology-dependent repair (HDR) or non-homologous end joining (NHEJ). HDR requires an intact DNA template and enables the precise restoration of the lesion [19]. In contrast, in NHEJ, broken DNA strands are re-ligated in an error-prone manner leading to nucleotide insertions or deletions (indels) [19]. Therefore, CRISPR/Cas9-mediated NHEJ is a common approach to generate a KO in human cells [13,19].

The delivery of the CRISPR/Cas9 system into some cell types remains a technical challenge [19]. Whereas human cell lines can be forced to express the CRISPR/Cas9 system simple by plasmid transfection [21], primary human cells require more sophisticated techniques [19]. Human ASCs are slowly dividing primary stem/progenitor cells with a doubling time between 3–4 days that undergo replicative senescence after approximately 20 population doublings in cell culture dependent on experimental conditions and donor characteristics (age, BMI, others) [14,22]. Moreover, the ASCs undergo changes in high passages [9]. Lentiviral delivery systems are the method of choice to genetically modify ASCs due to the capability of lentiviruses to transduce dividing and non-dividing cells with a broad tropism and high efficiency [19]. The components of the CRISPR/Cas9 system can be delivered into the cells either using single vectors (i.e., Cas9 enzyme and gRNA encoded on different plasmids/viruses) or an all-in-one system (i.e., Cas9 enzyme and gRNA encoded on the same plasmid/virus) [19]. Due to the short replicative life span of human ASCs the all-in-one system is necessary for this cell type.

In the present report, we provide insights into state-of-the-art CRISPR/Cas9 mediated gene knockout in human ASCs. Exemplifying this approach on the human *SPRY1* gene we demonstrate the efficacy of different CRISPR/Cas9 target sequences on the genomic, mRNA, protein and functional level. The presented technical procedures can be easily applied to other GOIs to study effects on ASC biology.

## Materials and methods

### Ethics declaration and donor characteristics

Subcutaneous white adipose tissue (sWAT) samples were obtained from patients undergoing routine elective plastic abdominal surgery at the Institute for Plastic and Reconstructive Surgery (Medical University of Innsbruck, Austria). All patients gave their informed written consent. The study protocol was approved by the Ethics Committee of the Medical University of Innsbruck (Austria) according to the Declaration of Helsinki. For this study, sWAT samples of n = 4 healthy donors of Caucasian origin were used (Supplementary Table 1).

### Isolation and culture of human adipogenic stem/progenitor cells (ASCs)

ASCs were isolated and maintained as previously described [23]. Briefly, sWAT samples were handled under sterile conditions, washed twice with PBS and dissected. Blood vessels, connective tissue and necrotic areas were removed. Subsequently, sWAT was enzymatically digested using collagenase I (200 U/ml in 2%BSA/PBS) for 1.5 h at 37°C while stirring. Afterwards, the mixture was filtrated using a strainer and centrifuged (10 min, 300 g, room temperature (RT)). The supernatant was removed and the pellet (=Stromal vascular fraction; SVF) was suspended in 30 ml Erythrocytes-Lysisbuffer (155 mM NH_4_Cl, 5.7 mM K_2_HPO_4_, 0.1 mM EDTA, pH 7.3) followed by filtration using a cell-strainer (100 µm pore size). Next, SVF cells were centrifuged (10 min, 300 g, RT), resuspended in 10 ml ASC2 medium (DMEM/F-12 (Gibco), 33 µM Biotin, 17 µM Pantothenate, 20 µg/ml Ciprofloxacin, 10% FCS) and filtrated using a cell-strainer (pore size 35 µm). Subsequently, SVF cells were seeded in 6-well plates at a density of 2 × 10^6^ cells/well. The supernatant was removed on the next day and replaced with serum-free ASC1 medium (same as ASC2 but without FCS). SVF cells were cultured for 6 days in ASC1 medium, harvested by trypsinization and seeded in 175cm^2^ cell culture flasks at a density of 5000–8000 cells/cm^2^ using ASC2 medium. On the next day, the supernatant was replaced by PM4 medium (ASC1 medium supplemented with 2.5% FCS, 10 ng/ml EGF, 1 ng/ml bFGF, 500 ng/ml Insulin). ASCs were splitted at a confluence of ~70%. ASC2 medium was used for seeding and replaced by PM4 medium for maintenance.

### Plasmid constructions

CRISPR/Cas9 plasmids were generated as described in the *Genome-Scale CRISPR Knock-out* (GeCKO) protocol (Supplementary Table 2) [20,24]. The plasmid pLentiCRISPR.v2 (Addgene #52961), which also contains a Puromycin-resistance cassette, was BsmBI digested to remove the filler fragment (1.89kb) and gel purified. Complementary DNA oligonucleotides containing selected CRISPR/Cas9 target sequences (Supplementary Table 3) were phosphorylated, annealed and ligated into the vector. Subsequently, plasmids were transformed into *E. coli Stbl3* by heat-shock and colonies were screened by PCR (Supplemental Procedures). Positive clones were propagated followed by plasmid purification using the EndoFree Plasmid MaxiKit (Qiagen) or the GeneJET Endo-free Plasmid Maxiprep Kit (Thermo Scientific) according to the supplier´s protocol. Next, plasmids were confirmed by analytical digestions (BamHI: linearization of plasmid yielding one 12.98kb fragment; BamHI/EcoRV: yielding one 10.66kb and one 2.32kb fragment) and sequencing. Final plasmids are designated as pLentiCRISPR.v2-GFP, pLentiCRISPR.v2-SPRY1#1 and pLentiCRISPR.v2-SPRY1#2. pLentiCRISPR.v2-GFP and pLentiCRISPR.v2-SPRY1#1 were already employed in our previous study [13]. shRNA-encoding vectors required for *SPRY1* gene knockdown were previously described [13]. A non-targeting shRNA was employed for comparison [10].

### Production of lentiviral particles and transduction of ASCs

Lentiviral particles were produced and titrated as described in detail in the Supplemental Procedures. ASCs were infected using a MOI of 4 followed by Puromycin selection (2 µg/ml) for 3–5 days.

### Differentiation of ASCs

ASCs were differentiated into adipocytes as described in Ref [11]. Briefly, ASCs were seeded in 6-well plates using ASC2 medium at a density of 10.000 cells/cm^2^. On the next day, the supernatant was replaced by PM4 medium and ASCs were cultured until confluence was reached. ASCs were serum starved (ASC1 medium) for 2–3 days followed by the induction of adipogenesis using differentiation medium (ASC1 supplemented with 0.2 µM Insulin, 0.25 µM Dexamethasone, 2.5% FCS, 10 µg/ml Transferrin and 0.5 mM IBMX). On day 3, the supernatant was replaced with fresh differentiation medium without IBMX. Thereafter, the medium was changed every 2–3 days.

### Generation of the U2OS-GFP-LC3 reporter cell line

U2OS human osteosarcoma cells were obtained from ATCC (USA) and transduced with a pLenti-GFP-LC3 overexpression construct under the control of the CMV promoter followed by Blasticidin selection. Cells were maintained in DMEM medium supplemented with 10% (v/v) FCS, 1% (v/v) Penicillin-Streptomycin and 1% (v/v) L-Glutamine.

### GFP reporter assay

The day before transfection, U2OS-GFP-LC3 cells were seeded into 6 – well plates with a density of ~ 0.5 × 10^5^ cells per cm^2^. Transfection with CRISPR/Cas9 plasmids (2 µg/well) was done using Lipofectamine2000 (ThermoScientific) according to the manufacturer´s protocol followed by Puromycin (0.6 µg/ml) selection. GFP fluorescence was measured using a FACS Canto II (BD Bioscience) instrument.

### Mutation detection assay

To determine CRISPR/Cas9-mediated mutations within the *SPRY1* gene, the PCR-based Guide-it Mutation Detection Kit (Takara Bio USA, Inc.) was used in accordance with the supplier´s protocol and as previously described in detail [13]. Cleaved DNA fragments were dissolved onto a 2% Agarose/TAE gel containing Midori Green and visualized using a Molecular Imager Gel Doc XR+ System (BioRad). Quantification of band intensities was done with Image Lab Software (Version 6.0.1; BioRad). Indel frequencies were calculated according to the formula described in Ref [25].: %Indel = 100 x [1-(1-fraction cleaved)^(1/2)^] fraction cleaved = (density of digested products)/(density of digested products + undigested parental band)

### Tracking of indels by decomposition (TIDE) analysis

Target-specific PCR products generated for the Mutation detection assay described above were Sanger sequenced using the forward primer and chromatogram files obtained were analysed with the TIDE webtool (https://tide.nki.nl/) [26,27].

### Gene expression analysis

Analysis of mRNA expression by RT-qPCR was done exactly as described [13]. PCR amplification was measured using SYBR green chemistry and the QuantStudio™ 7 Real-Time PCR system (AppliedBiosystems). Relative quantification of gene expression was calculated using β-Actin (*ACTB*) as endogenous reference and compared to the appropriate control sample as indicated (ΔΔC_T_ method).

### Western blot analysis

Western blotting was performed exactly as described in our previous study [13]. The anti-Sprouty1 antibody (Cell Signalling Technologies, #13013) was diluted 1:1000 and applied overnight at 4°C. β-Actin served as loading control. The anti-β-Actin antibody (SigmaAldrich; A5441) was used at a concentration of 1:100.000 and applied for 1 h at room temperature. Subsequently, appropriate secondary HRP-conjugated antibodies were employed followed by signal development with ECL. Detection of chemiluminescence was achieved using a ChemiDoc Imaging system (BioRad) and quantified with Image Lab Software (Version 6.0.1; BioRad).

### Quantification of intracellular lipids

ASCs were washed with PBS, fixed with 4% Paraformaldehyde (PFA)/PBS for 1 h at room temperature and washed again with PBS. Intracellular lipids were stained with Oil Red O (ORO) (see Supplemental Procedures) for 1 h at room temperature and washed twice with dH_2_O. Plates were air-dried and microphotographs (at least five per condition) were taken at randomly selected fields followed by counting of ORO stained cells. In addition, ORO was extracted with 1 ml/well Isopropanol for 30 min and absorbance was read at 570 nm.

### Statistical analysis

Statistics were performed using GraphPad Prism 5 software (GraphPad Software Inc., USA). Each set of experiments was conducted with a minimum of n = 3 biological replicates (i.e., donors). All measurements were done in three technical replicates. Values are provided as mean ± SEM. Statistical comparison was achieved with the two-tailed unpaired or paired *t*-test depending on the type of the data set and as mentioned in the corresponding figure legend. p values ≤ 0.05 were considered to be significant.

## Results

### Selection of CRISPR/Cas9 target sequences and vector construction

**Figure 1.**
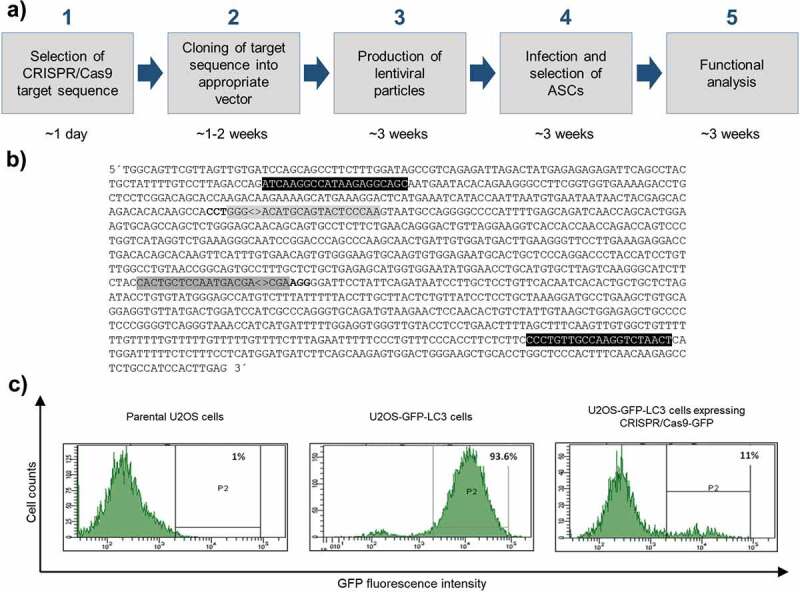
Overview of the experimental design of CRISPR/Cas9 experiments and proof of principle. (a) Work flow depicting all steps from CRISPR/Cas9 target sequence selection to downstream analysis and approximated time frame. (b) Partial sequence of the human *SPRY1* gene (123401618–123402790 Homo sapiens chromosome 4). Only the forward strand is shown. CRISPR/Cas9 target sequences are labelled in grey (#1: dark grey; #2: light grey). PAM sequences are depicted in bold letters. Note: the PAM sequence required for target #2 is located on the complementary strand. ‘<>’ indicates the *Streptococcus pyogenes* CRISPR/Cas9 cleavage site 3bp upstream of the corresponding PAM sequence. Primer sites for PCR amplification of target sites are shown in black. (c) Determination of the CRISPR/Cas9-GFP KO efficiency in U2OS cells stably expressing a GFP-LC3 fusion protein (U2OS-GFP-LC3) by FACS analysis. Intensity of GFP fluorescence was measured as readout. Left panel: FACS histogram of parental untransfected U2OS cells (no GFP expression). Middle panel: FACS histogram of U2OS-GFP-LC3 cells transfected with 8 μg DNA of a non-GFP targeting CRISPR/Cas9 plasmid (negative control) after Puromycin selection. Right panel: FACS histogram of U2OS-GFP-LC3 cells transfected with 8 μg DNA of the pLentiCRISPR.v2-GFP expression vector after Puromycin selection

To establish a *SPRY1* loss-of-function phenotype in ASCs we used the all-in-one lentiviral vector pLentiCRISPR.v2 for the expression of the entire CRISPR/Cas9 machinery [13,24]. The experimental design is outlined in Figure 1(a). Target sequences to generate a *SPRY1* knockout (KO) were retrieved from the GeCKO library [20,24] (Figure 1(b)) and inserted into pLentiCRISPR.v2. To generate a negative control, a CRISPR/Cas9 target sequence against the non-human Enhanced Green Fluorescent Protein (GFP) was used due to the absence of known off-target effects [20,28,29]. Other freely-available algorithms exist which will also provide CRISPR/Cas9 targets for a given gene of interest (GOI) (Supplementary Table 2). The CRISPR/Cas9 target sequences against *SPRY1* will be incorporated into the expressed gRNA and enable the CRISPR/Cas9 machinery to find its target site within the genome. Note, a PAM must be located adjacent of the genomic counterpart.

### Proof of principle

To evaluate whether the constructed all-in-one lentiviral CRISPR/Cas9 expression vectors mediate the degradation of a given target, we transiently transfected the pLentiCRISPR.v2-GFP plasmid into a U2OS reporter cell line stably expressing a GFP-LC3 fusion protein. As shown in Figure 1(c), CRISPR/Cas9-mediated targeting of GFP abolished the corresponding fluorescence signal demonstrating the power of the system.

### Generation of the lentiviral particles

Next, the endotoxin-free plasmids pLentiCRISPR.v2-GFP, pLentiCRISPR.v2-SPRY1#1 and pLentiCRISPR.v2-SPRY1#2 were co-transfected with helper plasmids (encoding components required for viral packaging and envelope) into the HEK293FT packaging cell line, harvested and titrated. An average concentration of n = 3 lentiviral preparations is given in Table 1. A high virus titre in the range of 1–3 × 10^6^ TU/ml was usually obtained despite the very large size of the pLentiCRISPR.v2 plasmid (~13kb). No statistically significant differences regarding yield were observed between CRISPR-GFP, CRISPR-SPRY1 #1 and #2.

**Table 1.** Average virus titre of n = 3 different charges. No statistically significant differences were observed between groups (Two-way Unpaired t-test). TU: transforming units

Lentivirus	TU/ml (± SEM)
CRISPR-GFP	3.77x10^6^ ± 1.16x10^6^
CRISPR-SPRY1 #1	3.10x10^6^ ± 1.28x10^6^
CRISPR-SPRY1 #4	1.75x10^6^ ± 2.9x10^5^

### Determination of CRISPR/Cas9 mutation efficiency

**Figure 2.**
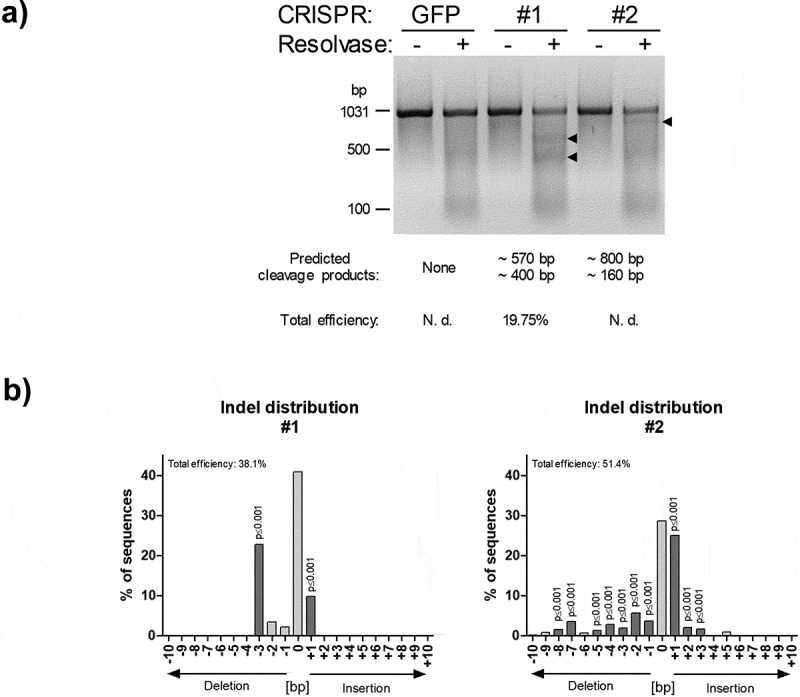
PCR-based analysis of *SPRY1* gene mutations. (a) PCR-based mutation detection assay according to the protocol provided by the *Guide-it Mutation Detection Kit* from *Takara Bio Inc*. (USA): The genomic region covering the expected CRISPR/Cas9-mediated mutations within the *SPRY1* gene was PCR-amplified (see, Figure 1(b)) in CRISPR/Cas9-GFP (GFP), CRISPR/Cas9-SPRY1 #1 (#1) and CRISPR/Cas9-SPRY1 #2 (#2) expressing ASCs. The PCR product (972bp) obtained from each cell population was melted and re-hybridized. This step results in the formation of DNA heteroduplexes originating from wildtype and mutated cells. Subsequently, mismatched DNA strands were cleaved by Resolvase and analysed by agarose gel electrophoreses. Predicted cleavage products are indicated by arrows. CRISPR/Cas9 efficiency was calculated as described in the methods section. N. d.: not determined; (b) Tracking of indels by decomposition (TIDE). The PCR product obtained in a was purified and Sanger sequenced using the forward primer. Distribution of indels within the targeted *SPRY1* gene locus was calculated using the TIDE algorithm as described in the methods section. A representative result of n = 4 donors is shown

To analyse the rate of mutations caused by NHEJ-mediated repair of CRISPR/Cas9 cleaved DNA, we used the PCR-based *Guide-it Mutation Detection Kit* from *Takara Bio Inc. (USA)*. Agarose gel analysis revealed the successful mutation of the intendent *SPRY1* gene locus by both gRNAs used (Figure 2(a)). To investigate the type of mutations in more detail, the PCR product (i.e., the PCR-amplified genomic locus) was subjected to Sanger sequencing and TIDE analysis. As shown in Figure 2(b), CRISPR-SPRY1 #1 and -#2 expressing ASCs showed a different distribution of indels corresponding with different efficiencies. In summary, these results demonstrate the capability of the employed CRISPR/Cas9 system to mutate the intended *SPRY1* locus as evidenced by qualitative and quantitative analysis.

### Depletion of Sprouty1 by CRISPR/Cas9 and shRNA

**Figure 3.**
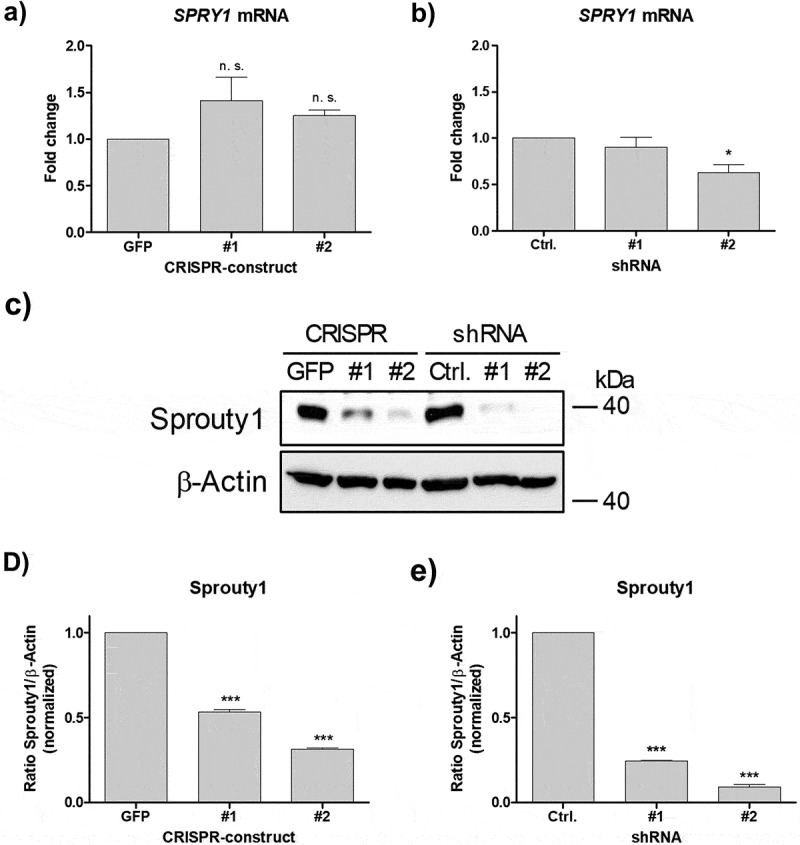
Effects of CRISPR/Cas9-SPRY1 and shSPRY1 on *SPRY1* mRNA and protein levels. RT-qPCR analysis of *SPRY1* mRNA levels in (a) CRISPR/Cas9 expressing ASCs and (b) ASCs transduced with appropriate shRNA constructs. Values are presented as mean ± SEM of n = 3 replicates. Statistical comparison was achieved using the Two-tailed paired t-test. (c) Western blot analysis corresponding to the results shown in A) and B). β-Actin served as loading control. (d and e): Densitometric analysis of the Western blot shown in C). Values are presented as mean ± SEM of n = 3 replicates. Statistical comparison was achieved using the Two-tailed paired t-test. Data obtained in CRISPR/Cas9 ASCs correspond to the results presented in Figure 2. A representative result of n = 4 donors is shown

Next, we tested whether CRISPR/Cas9 mediated mutation of the *SPRY1* gene affects mRNA synthesis. As shown in Figure 3(a), no significant effects on mRNA level were detected. For comparison, ASCs were transduced with two specific shRNAs targeting *SPRY1* mRNA as shown previously [12,13]. RT-qPCR analysis revealed a partial decrease of *SPRY1* mRNA expression in shSPRY1 ASCs (Figure 3(b)). Western blotting demonstrated a considerable depletion of Sprouty1 protein by both techniques employed thereby confirming a successful knockout (KO) and knockdown (KD) (Figure 3(c-e)).

### CRISPR/Cas9-mediated KO and shRNA-mediated KD of SPRY1 impairs adipogenic differentiation to a similar extend

**Figure 4.**
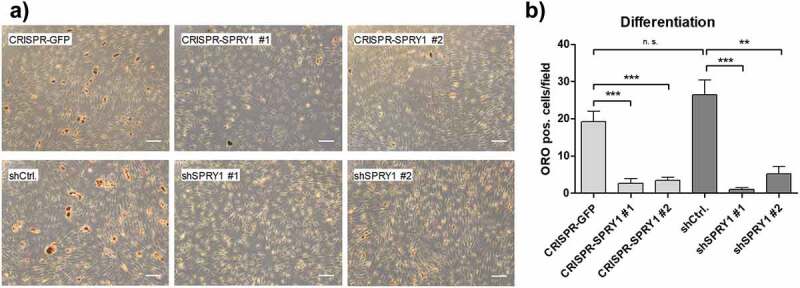
Oil Red O (ORO) staining of differentiated *SPRY1* KO and KD ASCs on d14. CRISPR-GFP and shCtrl ASCs served as negative controls. (a) Representative images of one donor out of n = 4 different experiments (i.e., donors) are shown. Magnification 50x. Scale bar 200 µm. (b) Quantification of adipocytes corresponding to A). n = 5 images per condition were taken randomly and ORO positive cells were counted. Values are presented as mean ± SEM. Statistical comparison was done using the Two-tailed Unpaired t-test. All results correspond to the data presented in Figures 2 and 3 and were obtained in the same donor

Finally, we elucidated whether *SPRY1* KO or – KD might be superior over the other in functional analysis. To this end, KO and KD ASCs were differentiated and mature adipocytes were quantified by Oil Red O (ORO) staining on d14. Reduction of Sprouty1 by both methods inhibited adipogenic differentiation as published previously [12,13]. Suppression of adipogenesis was achieved to a similar extend in *SPRY1* KO and KD ASCs (Figure 4). A comparison of *SPRY1* KO experiments among different donors is shown in Supplementary Figure S1. Taken together, despite different mechanisms CRISPR/Cas9 and shRNA are equally effective tools to study the function of a GOI in ASCs.

## Discussion

CRISPR/Cas9 genome-editing has become a powerful technique to study disease mechanisms in stem cells and other cell types [16]. Recently, we identified Sprouty1 (*SPRY1*) as a weight-loss target gene in human ASCs [12,13]. By employing lentiviral vectors, we could knockdown and knockout the *SPRY1* gene in human ASCs. These studies revealed that Sprouty1 is mandatory to maintain ASC proliferation and differentiation capacity and to prevent cellular senescence. In the present technical report, we provide comprehensive insights into CRISPR/Cas9-mediated gene KO in human ASCs using a single vector system encoding both the Cas9 endonuclease and the experimentally modified gRNA (all-in-one CRISPR/Cas9 system). We quantitatively assess *SPRY1* KO efficiency on gDNA using a mutation detection assay and TIDE analysis followed by a comparison to corresponding effects on mRNA and protein levels and functionality.

CRISPR/Cas9 efficacy is a critical issue dependent on the target gene, cell type and way of delivery [17,30]. Genome editing was so far mainly done in immortalized or transformed cell lines [31] and an overall efficiency up to ~80% has been reported [17]. For instance, *Hu et al*. used the human SGBS pre-adipocyte cell line for CRISPR/Cas9-mediated gene correction due to its higher susceptibility to genome editing compared to patient-derived ASCs [32]. However, performing such experiments in primary human cells, such as ASCs, is a technical challenge [31]. One reason is the limited proliferation capacity of ASCs which undergo replicative senescence soon after propagating in cell culture [9,14,22]. Therefore, selection of ASCs for a long time to reach a very high KO efficiency is not feasible. Moreover, loss of *SPRY1* triggers oncogene-induced senescence in this cell type leading to immediate cell cycle arrest after infection with the *SPRY1* KO lentivirus [13].

Our results show that CRISPR/Cas9 mutation efficiency is also determined by the target sequence used. Targeting the *SPRY1* gene by CRISPR/Cas9 target #1 was highly effective in all donors tested whereas the outcome of target #2 varied. Other target sequences showed no effect at all (data not shown). This observation indicates that also donor-dependent effects need to be taken into consideration when performing CRISPR/Cas9 genome editing in human ASCs. In an important study performed by *Lessard et al*., 2017 [33] the authors investigated the effects of human genetic variations on CRISPR/Cas9 specificity [33]. Herein it was shown that single-nucleotide polymorphisms (SNP) and indels can reduce on-target CRISPR/Cas9 activity.

The observed CRISPR/Cas9-mediated effects on the *SPRY1* gDNA did not prevent mRNA synthesis. Previously it was shown that frame-shift mutations as a consequence of NHEJ DNA repair can, but need not, result in a nonsense-mediated mRNA decay [34]. Therefore, the transcript level of a frame-shifted gene is not predictive for the corresponding protein level [34]. In line with this notion, Sprouty1 protein was considerably depleted by both CRISPR/Cas9 gRNAs employed thereby confirming the lack of a functional gene product (i.e., knockout). In addition, we recognized that the genomic *SPRY1* mutation rate measured by TIDE was below the rate of Sprouty1 protein depletion determined by Western blotting. One possibility might be a different susceptibility of both *SPRY1* alleles due to epigenetic mechanisms. Indeed, certain chromatin conformations can block the accessibility of the Cas9 enzyme to intended targets [35]. Studies have shown that modifying the chromatin by several approaches can increase CRISPR/Cas9 activity [35].

CRISPR/Cas9 off-target effects need to be considered and are mainly caused by mismatched gRNA:DNA interactions [16]. In addition, human genetic variations can contribute to an increased off-target frequency [33]. The incidence of unwanted editing events depends on the GOI and the cell type [16]. It was demonstrated that CRISPR/Cas9 off-target effects are very rare in healthy human cells [36]. Using whole-genome sequencing, *Veres et al*. detected only two to five unwanted CRISPR/Cas9-mediated events in individual human pluripotent stem cell (hPSC) clones [37]. The authors concluded that CRISPR/Cas9 does not cause a large degree of non-specific mutagenesis across the genome [37]. In our present study, we employed a non-targeting GFP-derived gRNA as negative control, which has also been used by others due to the absence of known counterparts within the human genome [13,20,28,29]. Moreover, in our study, all experiments were repeated at least three times using ASCs isolated from different donors to ensure reliable observations.

We compared the CRISPR/Cas9-mediated *SPRY1* KO and the shRNA-mediated *SPRY1* KD in ASCs derived from the same donors. Despite different mechanisms [38] both approaches successfully depleted Sprouty1 protein suggesting that these methods can complement each other. Thus, such a *modus operandi* could be helpful to corroborate functional results in given KD or KO experiments. The CRISPR/Cas9 sequences and shRNAs targeting *SPRY1* used in our technical report are the outcome of an extensive evaluation process. As expected, we observed that neither each single gRNA nor shRNA was equally effective to deplete Sprouty1 in ASCs from all donors examined (data not shown). Thus, no conclusion can be drawn whether one method is generally superior over the other. A careful experimental testing of several candidate sequences (i.e., genomic CRISPR/Cas9 target sites or shRNA sequences) for the efficiency of the down-regulation of a given GOI is highly recommended. Noteworthy, the combination of two or more shRNAs or gRNAs is a common approach to achieve a high rate of depletion [39]. A combined KD and KO within the same cell population has also been described [38]. We were capable to profoundly decrease the amount of Sprouty1 protein in human ASCs using a single gRNA and a single shRNA in independent experiments. This approach might be preferable due to a limited risk of off-target effects.

The current technical report describes a reliable CRISPR/Cas9-mediated KO of a GOI in human ASCs. Employing the all-in-one CRISPR/Cas9 system in association with high lentiviral transduction efficiency, followed by short-term Puromycin selection, a high KO efficiency and depletion of Sprouty1 protein was achieved. This robust approach could be applied to the down-regulation of other GOIs in human ASCs and other primary cell types.AcknowledgmentsThis study received intramural funding from the University of Innsbruck granted to W. Z.


## Supplementary Material

Supplemental Material
