# Supplementary material for: CRISPR/Cas9-mediated gene knockout in human adipose stem/progenitor cells
Source: Adipocyte. 2020 Oct 19;9(1):626–35. doi: 10.1080/21623945.2020.1834230 (PMC7575003; doi:10.1080/21623945.2020.1834230)
Supplement: Supplemental Material [file KADI_A_1834230_SM9398.zip › Supplementary figure legends.docx]

**Supplementary figure legends**

**Supplementary Figure S1:** Quantification of Oil Red O staining of differentiated CRISPR/Cas9-expressing ASCs by dye extraction of n=3 donors on d14. Values are presented as mean +/- SEM. Statistical comparison was done using One-way ANOVA and Dunett´s Multiple Comparison test.
