# Supplementary figures and images for: CRISPR/Cas9-mediated gene knockout in human adipose stem/progenitor cells
Source: Adipocyte. 2020 Oct 19;9(1):626–35. doi: 10.1080/21623945.2020.1834230 (PMC7575003; doi:10.1080/21623945.2020.1834230)

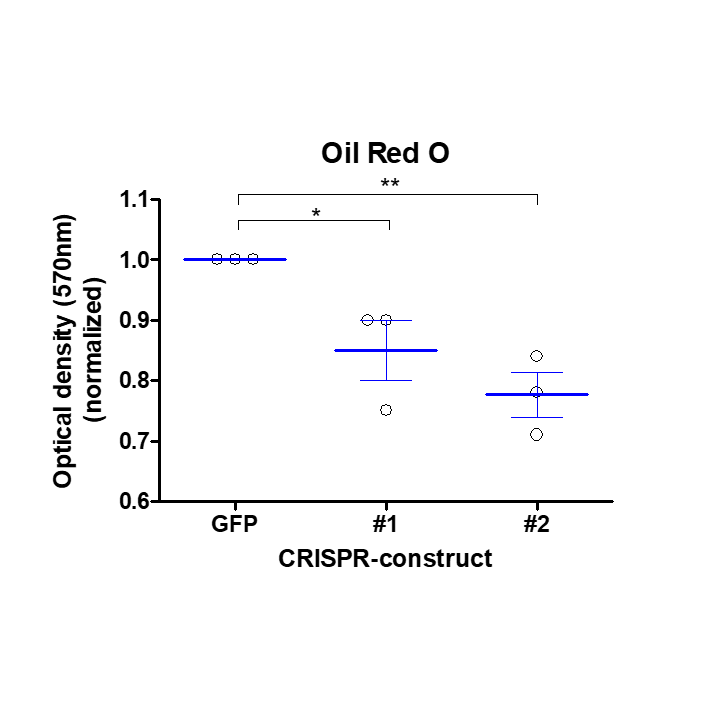

Supplement: Supplemental Material [file KADI_A_1834230_SM9398.zip › Supplementary_Figure_S1_ORO_OD_measurement_300DPI.tif]
